# Multiplexed Readout for an Experiment with a Large Number of Channels Using Single-Electron Sensitivity Skipper-CCDs

**DOI:** 10.3390/s22114308

**Published:** 2022-06-06

**Authors:** Claudio R. Chavez, Fernando Chierchie, Miguel Sofo-Haro, Jose Lipovetzky, Guillermo Fernandez-Moroni, Juan Estrada

**Affiliations:** 1 Fermi National Accelerator Laboratory, Batavia, IL 60510, USA; miguel.sofo@ib.edu.ar (M.S.-H.); gfmoroni@fnal.gov (G.F.-M.); estrada@fnal.gov (J.E.); 2 Departamento de Ingeniería Eléctrica y de Computadoras, Instituto de Investigaciones en Ingeniería Eléctrica “Alfredo C. Desages” (IIIE-CONICET), Universidad Nacional del Sur (UNS), Bahía Blanca 8000, Argentina; fernando.chierchie@uns.edu.ar; 3 Facultad de Ingeniería, Universidad Nacional de Asunción, Asunción 111421, Paraguay; 4 Centro Atómico Bariloche and Instituto Balseiro, Comisión Nacional de Energía Atómica (CNEA), Universidad Nacional de Cuyo (UNCUYO), Mendoza 5500, Argentina; lipo@ib.edu.ar

**Keywords:** Skipper-CCD, multiplexed readout electronics, CCDs, sub-electron counting, ultra low noise, analog charge pile-up

## Abstract

This paper presents the implementation of a multiplexed analog readout electronics system that can achieve single-electron counting using Skipper-CCDs with non-destructive readout. The proposed system allows the best performance of the sensors to be maintained, with sub-electron noise-level operation, while maintaining low-bandwidth data transfer, a minimum number of analog-to-digital converters (ADC) and low disk storage requirement with zero added multiplexing time, even for the simultaneous operation of thousands of channels. These features are possible with a combination of analog charge pile-up, sample and hold circuits and analog multiplexing. The implementation also aims to use the minimum number of components in circuits to keep compatibility with high-channel-density experiments using Skipper-CCDs for low-threshold particle detection applications. Performance details and experimental results using a sensor with 16 output stages are presented along with a review of the circuit design considerations.

## 1. Introduction

Silicon sensors are widely used as particle detectors [1,2]. Increasing the sensitivity of the experiments requires a larger sensitive mass to raise the total exposure (kg × time) and therefore improve detection probability. This imposes a challenge in terms of the number of readout channels, data bandwidth requirement, storage and noise performance of the sensors and electronics.

Specifically, charge-coupled devices (CCDs) are a type of silicon imager sensor composed of a matrix of pixels that collect ionized charge. They are widely used in instruments such as telescopes for astronomy and also as particle detectors due to their high sensitivity and spacial resolution. These types of sensors usually have a few readout video channels to read millions of pixels sequentially. The readout noise of a standard CCD is in the order of 2 or $3\;e^{-}$, mostly limited by the white and 1/f noise combination of the output amplifier of the sensor [3].

### Skipper CCD and LTA Electronics

The Skipper-CCD [4,5] is a CCD with a modified, non-destructive, readout output stage that allows one to measure the charge of each pixel several times. Averaging the independent charge measurements results in sub-electron noise levels, turning the Skipper-CCD into a photon-counter sensor. There are also other types of non-destructive imaging sensors [6,7]. A specific readout electronics system, the low-threshold acquisition controller (LTA), was designed to control and process [8,9] the Skipper-CCD signals [10]. This system has achieved the best performance with the sensor, but due to its fully digital, high-speed video signal sampling and processing, its architecture could not be easily extended for experiments that feature kilograms of Skipper-CCDs with thousands of video channels as sensitive mass.

For smaller experiments, the good performance obtained by the Skipper-CCD and the LTA, achieving sub-electron noise with a wide dynamic range, has promoted the progress and planning of many scientific experiments for particle detection.

The Coherent Neutrino-Nucleus Interaction Experiment (CONNIE) [11] searches for reactor neutrinos using standard CCD detectors and it is currently upgrading to Skipper-CCDs [12]. Moreover, the Neutrino Interaction Observation with a Low Energy Threshold Array (ν IOLETA experiment) is planning to use several kg of Skipper-CCDs [13] in a nuclear reactor.

The Sub-Electron-Noise Skipper-CCD Experimental Instrument (SENSEI) uses Skipper-CCDs for Light Dark Matter search [14] and the Dark Matter in CCDs experiment (DAMIC-M) [15] is also planning to do so.

In the same direction, the Observatory of Skipper CCDs Unveiling Recoiling Atoms (OSCURA experiment) is planning to combine the efforts of ongoing experiments to design and build the largest experiment with 10 kg of sensitive silicon mass and around 24,000 readout channels for dark matter detection based on Skipper-CCD [16]. The OSCURA detector is in the research and development stage, and in this paper, we present the experimental results of the multiplexed readout electronics designed for OSCURA.

The proposed system allows the best performance of the Skipper-CCD to be maintained, with sub-electron noise operation, while keeping low-bandwidth data transfer, a minimum number of analog-to-digital converters (ADC) and low disk storage requirement—all with zero added multiplexing time.

This paper is organized as follows. In Section 2, we briefly describe the characteristics of the OSCURA experiment and the requirements in terms of the readout system. A detailed description of the readout electronics is presented in Section 3. An experimental demonstration is presented in Section 4, where the performance of the electronics with the sensor is evaluated. Comparisons of two multiplexing strategies are performed: the standard readout, where the pixel values are multiplexed after computing the pixel value, and a novel parallel readout, where the pixel values are multiplexed in parallel, while computing the next pixel value. Finally, Section 5 presents the conclusions.

## 2. OSCURA Experiment

The OSCURA (Observatory of Skipper CCDs Unveiling Recoiling Atoms) [16] experiment is in its research and development stage for the development of a 10 kg Skipper-CCD experiment for low-mass dark matter (DM) search—specifically for DM particle candidates with masses below GeV (“sub-GeV DM”). The expected signal is induced by one or two ionized electrons released by the DM candidate interacting directly with the electrons in the Skipper-CCD detector.

Some of the main challenges that must be addressed for the experiment are:

- Sensors R&D: Skipper-CCDs require a special fabrication process due to their relatively high operation voltage and the silicon thickness required to increase the sensitive mass of the sensor. New sensors developed at Microchip Inc. are currently being tested and characterized.
- Background Radiation R&D: the scientific goal of the OSCURA project requires an extremely low background radiation. This implies a detailed understanding and mitigation of all the radioactive sources near the detectors, including fabrication materials in the electronic components, flex cables, etc. Background radiation is a problem in the sense that particles emerging from contaminated materials can mask in many ways the events with the characteristics of DM signals.
- Readout Electronics R&D: Given the standard dimensions of a Skipper-CCD pixel (15 µm × 15 µm) and considering the thickness (≈650 µm) of silicon wafers for thick CCDs, a detector with a sensitive mass of 10 kg will require 28 gigapixels. The number of output channels depends on the exact size chosen for individual sensors. For one of the candidates, a sensor with a size of 1058 × 1278 pixels, the number of video (output) channels needed is 24,000. The total readout time is also a key point, since a very long readout time results in an increased dark current count, which could mimic the DM signal. There is a trade-off between the readout time required by the experiment and the number of readout channels.

Among the challenges mentioned above, this paper focuses on the readout electronics. We present the preliminary experimental results of a novel multiplexed and scalable readout system that could achieve single-electron resolution. The circuit also reduces the data throughput by measuring the skipper samples using a charge pile-up technique, which allows only the final pixel value with reduced noise to be sent to the analog-to-digital conversion (ADC) stage. The reduction in data rate, along with the multiplexer, allows the reading of up to 6400 video channels with the same single ADC that was previously used to read a single fully digital video channel [10].

**Figure 1:**
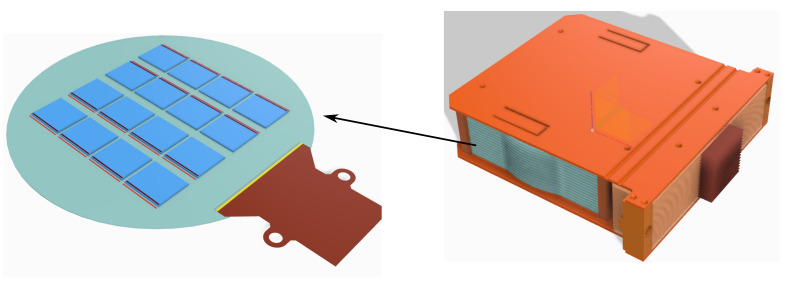
(**Left**) Design of the OSCURA Multi-Chip Module (MCM) with 16 sensors mounted on a 150 mm silicon wafer; (**Right**) OSCURA super-module (SM) with 16 MCMs held by a copper support structure.

The left-hand drawing in Figure 1 shows the conceptual design of the Multi-CCD Module (MCM) with 16 Skipper-CCDs mounted on a 150 mm silicon wafer that also has a flex cable used to route the clocks of the CCDs and the 16 video channels of the MCM. On the right, a super-module (SM) features 16 MCMs (a total of 256 Skipper-CCDs) held by a copper support structure.

**Figure 2:**
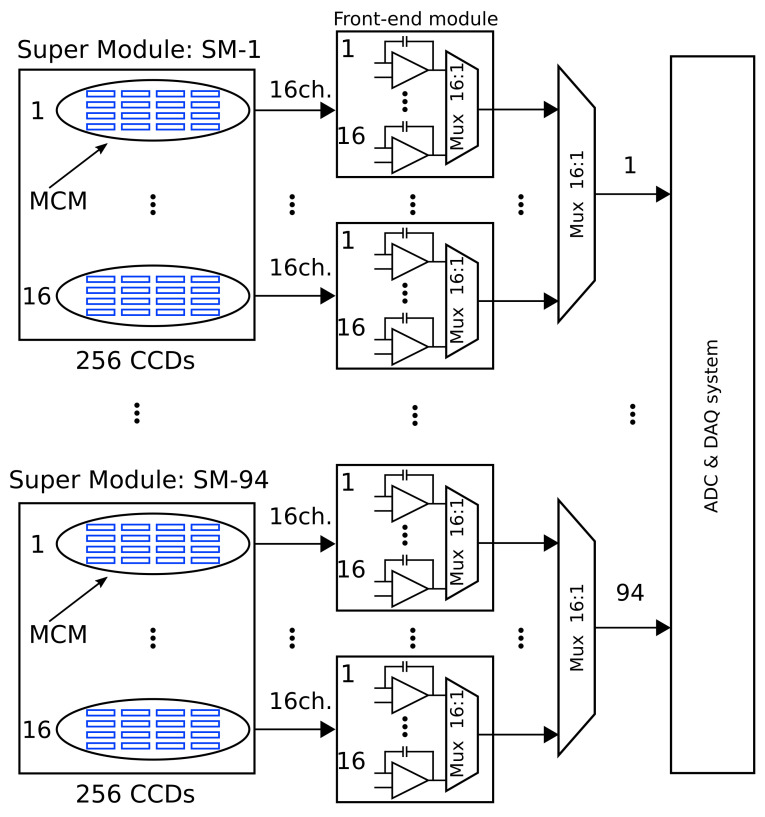
Block diagram of the OSCURA system with two-stage multiplexing.

Figure 2 shows a block diagram of the whole OSCURA system, including the sensors, multiplexed readout electronics and DAQ system. The experiment will have a total of 1500 MCMs and 24,000 video channels. Each of the 94 super-modules has 256 Skipper-CCDs, gathering a total sensitive silicon mass of around 10 kg. The readout architecture computes the pixel value and performs the skipper sampling averaging for each output. Then, it has a two-level multiplexing stage, where each MCM with 16 video channels is multiplexed into a single signal through the first-level multiplexers and then again through the second-level ones, resulting in one signal per SM. A total of 94 signals are finally acquired by the analog-to-digital converters (ADCs).

## 3. Description and Analysis of the Proposed Front-End Electronics

One of the key components in this high-density system, summarized in Figure 2, is the front-end electronics module and the multiplexing stage that follow the super-module array. These electronic modules are in charge of processing the low-level signals coming from each sensor and producing at their outputs a signal with a high signal to noise ratio compatible with two stages of analog multiplexing. The system should also be able to drive all the analog wiring without degrading the performance of the sensors before reaching the analog-to-digital converter (ADC). Low complexity and a low component count per channel are also required due to the high number of channels and minimum background radiation requirements. Packaging materials and printed circuit board (PCB) substrates are sources of radiation.

These front-end electronic modules must compute the pixel value using the analog processing chain proposed in [17] with the addition of an S&H circuit, an analog multiplexer and a novel parallel readout scheme. This section describes in detail the operation of this chain combined with the multiplexing stage along with the mathematical equations that support both the operation and the impact on the requirement of experiments with large arrays of Skipper-CCDs.

The value of a pixel in a Skipper-CCD is given by [5]

(1) $$\begin{array}{c} P_{i}=\sum_{j=0}^{N-1}\left(\int_{t_{i}+\tau +t_{j}}^{2t_{i}+\tau +t_{j}}V_{CCD}\left(t\right)dt-\int_{t_{j}}^{t_{i}+t_{j}}V_{CCD}\left(t\right)dt\right), \end{array}$$

where $P_{i}$ is the current pixel value; $t_{j}$ is the initial time on each measurement of the pixel charge and can be computed as $t_{j}=j\left(2t_{i}+\tau +\psi \right)$, where ψ models any dead time between consecutive pixel measurements; τ is the time spent to transfer the charge to the sensing node after the reset, and $t_{i}$ is the pixel integration time and $V_{CCD}\left(t\right)$ the video signal produced by a CCD. *N* represents the number of times a single charge packet is measured. The averaging factor 1/N and other scaling gains were omitted in Equation (1) for simplicity.

This equation can be seen as consecutive double slope integrations (DSI), where the initial value of the current integration is the final result of the previous integration. For the first integration, the initial value is 0, and the result of the last integration represents the current pixel value. This is what the analog processing chain must perform in order to obtain the value of a pixel. This processing chain can be also used as a base design for a custom Application-Specific Integrated Circuit (ASIC) that performs the same function.

The front-end electronic modules have three main parts: the analog processing chain, sample and hold circuit and first-stage multiplexer:

### 3.1. Analog Processing Chain

To compute Equation (1), the circuit uses two stages: the pre-amplifier and the re-configurable integrator.

**Figure 3:**
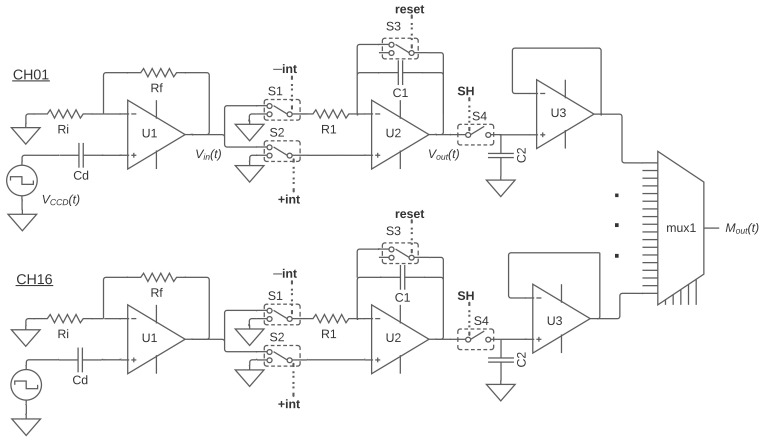
Simplified schematic of the analog processing chain and the first-stage multiplexer. Switch selection is shown for logic states **+int** = 1, **−int** = 1, **reset** = 1 and **SH** = 0.

The pre-amplifier U1 in Figure 3 provides a first stage with high input impedance to keep minimum loading to the CCD output stage and amplification gain. Since it is the first gain stage after the CCD, the low noise characteristic and gain improve the signal to noise ratio before feeding the rest of the electronic chain. This stage also filters the signal to limit the bandwidth to a maximum of a few hundreds of kHz. Rf and Ri set the gain of this stage.

The re-configurable integrator is implemented through U2 along with R1, C1, as shown in Figure 3. It acts either as an inverting integrator or a non-inverting integrator depending on the states of switches S1 and S2. It is a different implementation approach from the more common and complex combination of a fixed inverting integrator with a re-configurable inverter/non-inverter buffer [18,19], which requires additional switches and operational amplifiers.

**Figure 4:**
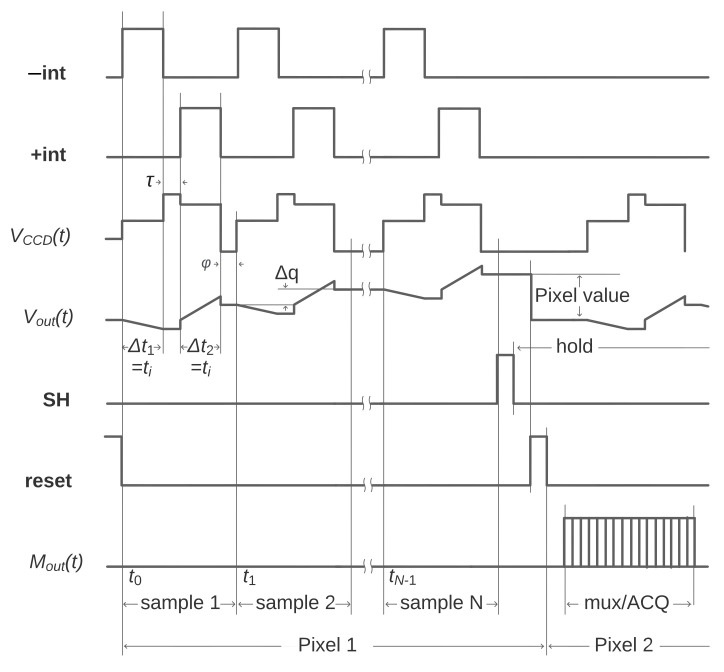
Simplified timing diagram of the acquisition sequence. $\Delta t_{1}=t_{i}$ and $\Delta t_{2}=t_{i}$ are the periods of inverting and non-inverting integration, respectively; Δq represents the increment from the previous DSI result. **−int**, **+int**, **SH** and **reset** are the digital control signals of the switches. $V_{CCD}\left(t\right)$ is the CCD video signal, $V_{out}\left(t\right)$ is the output of the integrator and $M_{out}\left(t\right)$ is the output of the analog multiplexer.

Three digital lines are required to configure and control the integrator, **+int**/**−int**, to select the non-inverting or inverting integration configuration through switches S1 and S2, and a reset control line for the switch S3. A timing diagram of the operation is shown in Figure 4; $V_{out}\left(t\right)$ represents the integrator output. The first dual-slope integration (DSI) starts when **−int** = 1 and **+int** = 0 during the interval $\Delta t_{1}=t_{i}$; S1 connects the pre-amplified video signal to the inverting input of the integrator and S2 connects the non-inverting input to the ground. This falls within the pedestal or reference time of the CCD output signal and sets the first slope of the DSI (inverting). When **−int** = 0 and **+int** =1 during the interval $\Delta t_{2}=t_{i}$, switches swap connections and set the integrator for the second slope of the DSI (non-inverting). This falls within the charge level time of the CCD output. The process is repeated *N* times and for each consecutive DSI; Δq represents a measurement of the charge packet and the increment from the previous DSI result (charge pile-up). After *N* iterations, **reset** = 1 produces a discharge of the integrating capacitor through S3 and the integrator is then ready for the next pixel. When **−int** = 0 and **+int** = 0, both inputs are grounded and the circuit holds the previous output. This happens during the dead times and during the time needed to transfer the charge to the sensing node; these are τ and ψ in Equation (1), respectively.

Note that during non-inverting integration, the configuration of the circuit is what is known as non-inverting integrator approximation. The output is given by

(2) $$\begin{array}{c} V_{out}\left(t\right)=\frac{1}{R1\times C1}\int_{0}^{t}V_{in}\left(t\right)dt+V_{in}\left(t\right), \end{array}$$

where $V_{out}\left(t\right)$ is the output of the integrator in the non-inverting configuration and $V_{in}\left(t\right)$ is the signal at the non-inverting input, both at time *t*. This means that the input is copied at the output and added to the current integration, causing a jump in the output signal when $V_{in}\left(t\right)$ is not connected to the ground. This does not affect the operation because, after the integration ends, both inputs (inverting and non-inverting) are connected to the ground (**+int** = 0, **−int** = 0). This sets $V_{in}\left(t\right)=0$ after the interval $\Delta t_{2}$ and the output then stays at the final value of the non-inverting integration, which is given by

(3) $$V_{out}=\frac{1}{R1\times C1}\int_{\Delta t_{2}}^{}V_{in}\left(t\right)dt,$$

where $V_{out}$ is the output of the integrator after the interval $\Delta t_{2}$. The presence of the input signal added to the output of the non-inverting integrator sets a condition for when the output must be evaluated, but the analog processing chain remains valid for computing Equation (1). This behavior during the non-inverting integration is illustrated in Figure 4 as a small jump during the $\Delta t_{2}$ interval in $V_{out}\left(t\right)$.

As shown in Figure 4, through the timing diagram of $V_{out}\left(t\right)$ (integrator output), the integrating capacitor C1 stores and piles up the intermediate pixel values. For each of the *N* independent samples of the same pixel, the capacitor accumulates a charge proportional to the charge generated in the current pixel. It only resets and goes back to zero at the beginning of a new pixel. Therefore, the voltage stored in the integrating capacitor at the end of the sequence is proportional to the charge of the current pixel.

The voltage at integrator output at the end of the *N* pixel samples is given by

(4) $$\begin{array}{c} V_{\mathrm{out}}\left(t\right)=\sum_{j=0}^{N-1}\frac{1}{R1\times C1}\int_{jT_{pix}}^{\left(j+1\right)T_{pix}}\left(S2_{out}\left(t\right)-S1_{out}\left(t\right)\right)dt, \end{array}$$

where $S2_{out}\left(t\right)$ and $S1_{out}\left(t\right)$ are the output of the selection switches S1 and S2; $T_{pix}=2t_{i}+\tau +\psi$ is the pixel period. These switches are connected to the same pre-amplified video signal but during different periods of time. S1 connects the video signal to its output precisely only during the reference (or pedestal) level, while S2 connects only during the charge level. As indicated by $\Delta t_{1}$ and $\Delta t_{2}$, respectively, in Figure 4, the remaining time switches S1 and S2 are connected to the ground. This is expressed by

(5) $$\begin{array}{ccc} S1\left(t\right)=\left\{\begin{array}{ccc} P & \mathrm{if} & t_{j}\leq t\leq t_{i}\;+\;t_{j}\\ 0 \end{array}\right. & \; & S2\left(t\right)=\left\{\begin{array}{ccc} P+Q & \mathrm{if} & t_{i}\;+\;\tau \;+\;t_{j}\leq t\leq 2t_{i}\;+\;\tau \;+\;t_{j}\\ 0 \end{array}\right. \end{array}$$

where *P* is the expected reference (pedestal) level and *Q* is the voltage increment produced by the charge packet in the current pixel. Using Equations (4) and (5), the signal produced by the integrator alone, for *N* samples of pile-up, is given by

(6) $$\begin{array}{c} V_{\mathrm{out}}=N\left(\frac{1}{R1\times C1}\right)t_{i}Q. \end{array}$$

The factor multiplying *Q* in Equation (6) is the gain of the re-configurable integrator. Taking also into account the gain of the pre-amplifier *A* and the sensitivity of the CCD sense node S≈2 µV/e ${}^{-}$, the gain of the processing chain at the end of the sequence is given by

(7) $$\begin{array}{c} G[V/e{}^{-}]=A\times S\times N\left(\frac{1}{R1\times C1}\right)t_{i}. \end{array}$$

### 3.2. Sample and Hold Circuit (S&H)

The sample and hold (S&H) circuit was added to provide an auxiliary storage capability for the front-end electronic module. Using C2, S4 and U3 as a buffer, it holds the final pixel value during the multiplexing and digital conversion. When **SH**= 1, S4 connects the integrator output to C2 briefly. After this, C2 is charged with the result of the last DSI of the sequence, which is the pixel value for our purposes. This happens right before resetting the integrator; the timing diagram is shown in Figure 4. The S&H circuit serves two purposes: avoiding the higher drift that integrators introduce while holding the final pixel value, and holding this final pixel value during multiplexing and digital conversion so that the processing chain can set its integrators to reset state and start a new pixel independently in parallel, as shown in Figure 4, where the second pixel starts at the same time that the multiplexing takes place.

### 3.3. First-Stage Multiplexer

The analog multiplexer mux1 in Figure 3 passes the signals of each S&H circuit from each channel to a single ADC in the acquisition system one by one. This happens after all the pixel values are already being held, as shown in Figure 4. The high signal to noise ratio archived by the combination of the Skipper-CCD and analog pile-up at the end of the sequence makes negligible the noise contribution of the S&H circuit and analog multiplexer. This makes this scheme compatible with experiments with high-density arrays of CCD sensors where the sub-electron noise capability and minimum added multiplexing time are required.

### 3.4. Analog Pile-Up Advantages Analysis

Analog piling up brings two advantages that make an important difference, especially in high-channel-density experiments such as OSCURA. These are the lower data throughput and transfer requirement. The three main methods for computing the pixel from a Skipper-CCD output signal are:

- Fully digital signal processing: uses only a pre-amplifier and then a high-speed ADC. The pixels are calculated digitally with a microprocessor or a Field-Programmable Gate Array (FPGA) and the *N* pixel samples are averaged digitally either in the electronics or in a computer.
- Mixed analog/digital DSI (double slope integration): this is the most common method for scientific CCDs, where each pixel is calculated using an analog DSI circuit and then an ADC digitizes the value. The pile-up is done digitally so each pixel sample must be sent and stored during readout.
- DSI + fully analog pile-up: the circuit proposed in [17] falls into this type and is used in this proposal with the added S&H and multiplexing. The whole DSI and analog pile-up is done by the analog circuit and the final pixel value is only digitized at the end of the last pixel sample. Data transfer and storage have the lowest requirement among the three methods described.

Popular scientific CCD controllers such as Leach [18] and Monsoom [19] use the mixed analog/digital DSI method to calculate the pixel value. The LTA [10] uses a high-speed ADC (15MSps) and computes the pixel value with a microprocessor. All these three systems transfer the digital pixel value as soon as they compute it. In the case of a Skipper-CCD, this means one transfer for every pixel sample. Therefore, the total amount of data $D_{\mathrm{image}}$ in bits that need to be transferred per megapixel of image from the front-end electronics to the DAQ computers or storage device is given by

(8) $$\begin{array}{c} D_{\mathrm{image}}=b_{\mathrm{pix}}\times N\times 10^{6}\;\left[\mathrm{bits}/\mathrm{Mpix}\right], \end{array}$$

where $b_{\mathrm{pix}}$ is the ADC resolution in bits and *N* is the number of samples per pixel.

Depending on how the DAQ computer processes each skipper pixel sample to obtain a final Skipper-CCD image, it could be necessary to buffer the data transferred before processing. This translates into a storage requirement, which is the case for the controllers cited above.

The data throughput at the ADC output per channel is given by

(9) $$\begin{array}{c} W=b_{\mathrm{pix}}f_{s}\;\left[\mathrm{bps}\right], \end{array}$$

where $f_{s}$ is the sampling frequency and $b_{\mathrm{pix}}$ is the ADC bit resolution. For the fully digital method, $f_{s}$ is equal to the continuous sampling frequency. For the other two common methods, the sampling frequency is equal to the pixel frequency $f_{\mathrm{pix}}=1/T_{pix}$, which is approximately equal to $1/(2t_{i})$. In the case of fully digital signal processing, the throughput is constant and independent of the pixel frequency since it is set by design. For the case of analog pile-up, the ADC only sends data every *N* skipper pixel samples, since, during pile-up, the the integrating capacitor is holding the intermediate pixel values. Therefore, data throughput from the ADC $W_{AP}$ and the amount of data to be transferred $D_{AP}$ are given by

(10) $$\begin{array}{ccc} W_{AP}=\frac{W}{N}\;\left[\mathrm{bps}\right] & \; & D_{AP}=\frac{D_{\mathrm{image}}}{N}\;\left[\mathrm{bits}/\mathrm{Mpix}\right], \end{array}$$

Table 1 compares the three methods in terms of data throughput from the ADCs and the amount of data that need to be transferred using Equations (8) and (9) for a 1500×9000 pixels Skipper-CCD, with $b_{\mathrm{pix}}=$ 18 bits per pixel and N=400 skipper samples per pixel.

As shown in Table 1, the analog pile-up technique reduces both the data throughput and amount of data by at least least 99% compared to other methods described. These reductions have critical impacts in a high-channel-density front-end.

## 4. Experimental Verification

**Figure 5:**
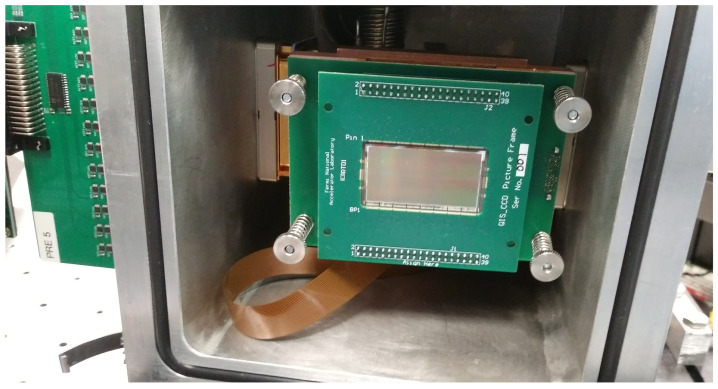
The 16-channel Skipper-CCD used for experimental verification in the chamber.

For design and concept verification, a test was performed at the Fermi National Accelerator Laboratory using a 16-channel Skipper-CCD with 4096×2048 pixels of (10 µm × 10 µm) each. A substrate bias was applied to fully deplete the substrate, which was 675 µm thick. The high resistivity, ≈20 k Ω· cm, allowed for fully depleted operation at a substrate voltage of around 70 V. The sensor was kept in a vacuum vessel at vacuum greater than $1\times 10^{-4}$ torr during readout and temperature was held at 140 K using a cryochiller and a closed loop controlled with a heater. Figure 5 shows the 16-channel Skipper-CCD installed inside the test chamber using a picture frame and connected through a low-thermal-conductivity flex cable to a vacuum feed-through connector out to the front-end electronics (partially seen at the left of the image).

**Figure 6:**
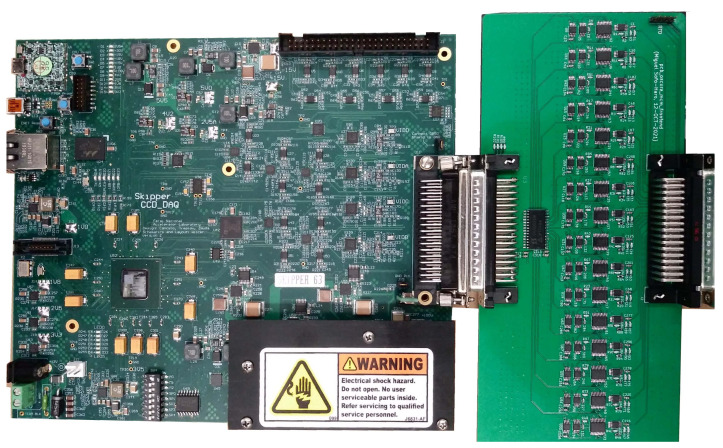
LTA connected to the 16-channel multiplexed analog front-end board.

The 16-channel multiplexed analog processing chain was connected between the Skipper-CCD and the LTA [10] as shown in Figure 6. The LTA was in charge of providing bias voltages and clocks for the Skipper-CCD, commanding the switches in the analog processing chain and digitizing the final pixel value through a single ADC. The LTA had an input range of ±1 V with a pre-amplifier and an 18-bit and 15 MSps ADC.

All the operational amplifiers used were Texas Instruments OPA140, the single pole double trow (SPDT) switches were Maxim Integrated MAX333A and the multiplexer was a Vishay DG506B. The pre-amplifier gain was set to 5 using Rf=1 k Ω and Ri=240Ω. Integration gain was set using C1=18 nF and R1= 2 k Ω. These values were selected to have a dynamic range of approximately 800 $e^{-}$ for N=400 and $t_{i}=16$ µs. The dynamic range increases with lower values of $t_{i}$ and *N*.

The main selection criteria for the operational amplifier were the noise characteristic (5.1 nV/$\sqrt{\mathrm{Hz}}$), its ability to operate with ±18 V power supplies and the input offset voltage. The noise characteristic has an impact on the total noise added by the front-end (base noise), and the offset voltages impact the drifting seen at the output of the integrator. The latter does not represent a problem for the image integrity, since it can be removed with standard CCD baseline subtraction procedures. However, any drift can reduce the dynamic range during long integration times (high *N*). The low noise criterion was also used for the switches and multiplexer selection along with switching speed requirements. Availability in bare-die version of the semiconductors was also a key selection criterion; a bare-die version of these circuits is being considered for the purpose of background radiation mitigation.

**Figure 7:**
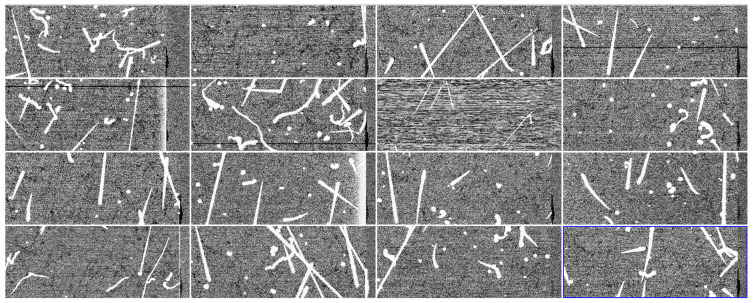
Sample images obtained for the 16 channels after software de-multiplexing. Cosmic ray events are observed.

Figure 7 shows a fraction of the 16 images captured, one for each channel, once de-multiplexed via software. The muon and electron tracks seen in the images show that the whole system is working and producing valid images as a starting point.

**Figure 8:**
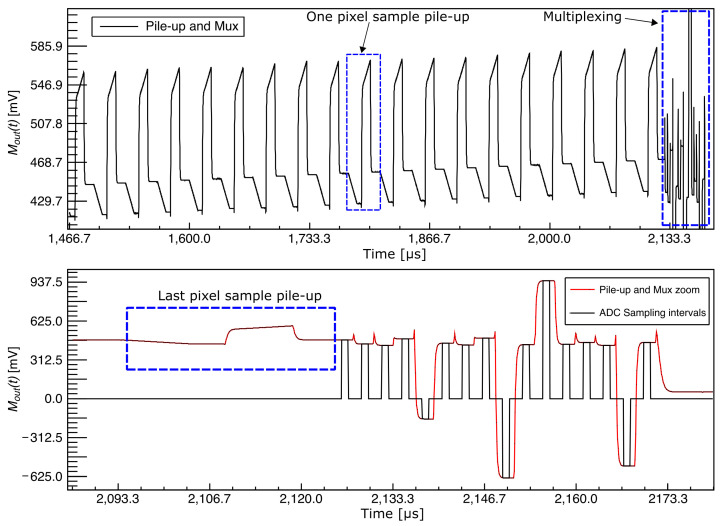
(**Top**) Raw signal measurement of the pile-up signal and multiplexer output. The sample of one pixel being measured and pile-up is indicated. The multiplexing is also shown; (**Bottom**) Zoomed-in region of the top figure including the last pixel sample pile-up and the 16-channel multiplexing.

The top plot in Figure 8 shows a capture of the raw signal at the output of the multiplexer. For this capture, the multiplexer was held connected and steady to one of its inputs during the analog pile-up, while the S&H circuit was disabled. This way, the pile-up for each pixel sample could be seen in the capture as a repetition of the shapes enclosed by the blue dashed square, where the final level of each repetition is higher than the previous one. When the pile-up ends, the integrators on each channel are holding the final pixel value for the current pixel of their channel, as explained in Section 3. The multiplexation then starts, as is shown in the last part of the top plot in Figure 8. The bottom plot shows a zoomed-in version where the multiplexer output is in red. The integrators of each channel are connected to the main output in sequence one by one through the multiplexer producing the 16 slots of approximately 3.1 µs, as shown in the plot. The sampling intervals shown in black are the portion of each slot where the LTA uses its high-speed ADC to digitize the final pixel value on each channel. These intervals are narrower than the channel slot because there is a transience every time the multiplexer switches between channels that must settle down before sampling. This avoids introducing unwanted noise from this transience to the final pixel value. Moreover, since only a few ADC samples are needed on each interval, the sampling interval stops before the end of the channel slots.

### 4.1. Sub-Electron Noise Measurements

**Figure 9:**
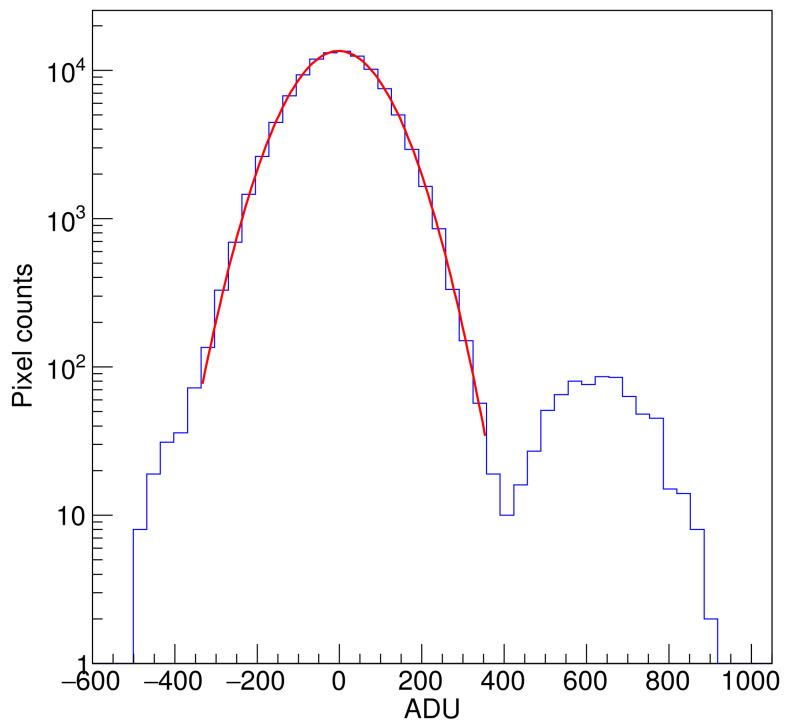
Histogram of pixels in overscan for one channel. A gain of 622$\;ADU/e^{-}$ and noise of 0.16$\;e_{rms}^{-}$ were obtained by fitting Gaussian functions to each of the peaks. Zero-electron peak fitting shown with red line.

Sub-electron noise levels were achieved as predicted using multiple samples per pixels. Figure 9 shows a histogram of pixel values from one channel using N=400 skipper samples per pixel and $t_{i}=16$ µs, in ADC units (ADU). The two peaks correspond to the distribution of pixels with 0 $e^{-}$ and 1 $e^{-}$ charge. Noise level was 0.16 $\;e_{rms}^{-}$ or 99.52 $ADU_{rms}$, producing a clear separation of the pixels with 0$\;e^{-}$ charge from the ones with 1$\;e^{-}$ charge.

**Figure 10:**
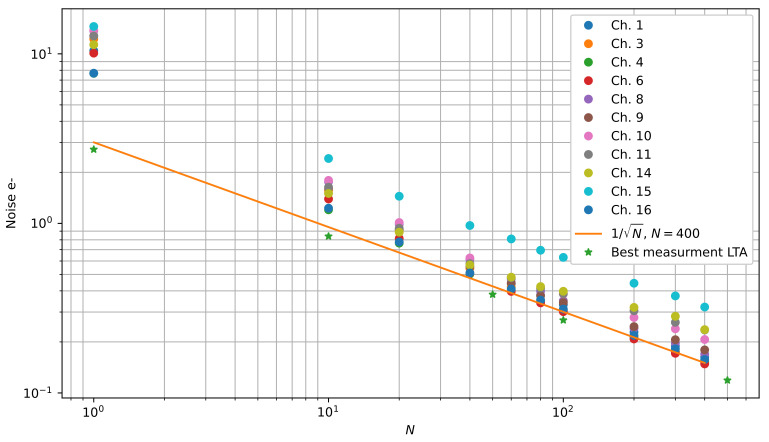
Noise versus number of samples per pixel (*N*). The stars show the best measurement with Skipper-CCD and LTA controller reported in [10]. Solid lines show the expected $1/\sqrt{N}$ reductions, taking a point at N=400 as the starting point.

Figure 10 shows a plot of the noise level versus the number of samples per pixel *N* for integration time $t_{i}=$16μs. The green stars correspond to the noise levels measured in [10] for a 4-channel Skipper-CCD with the fully digital LTA. The orange line shows the expected $1/\sqrt{N}$ rate of noise reduction, taking as a starting point the noise for the best measured channel at N=400. The different colored dots represent all the channels of the 16-channel sensor with sub-electron noise levels. The performance of the best channels was very close to what is shown in [10] for N≥40, but some channels exhibit higher noise levels. This is a common situation and it is the reason that a pre-test is needed to select the best detectors for an experiment. Note, however, that in this region, all the channels have the same noise reduction rate ($1/\sqrt{N}$ seen as an straight line in a log-log plot). This means that they follow a line parallel to the orange line. For a low number of samples per pixel (N<40), the gain is low according to Equation (7), and the chain struggles to obtain a good signal to noise ratio due to the circuits’ inherent noise sources and the quantization noise at the analog-to-digital conversion stage. At N=1, the output voltage of the processing chain produced by 1 $e^{-}$ in the pixel is around half of the resolution of the ADC (31.25 µV), so the quantization noise could well be dominating the performance. The gains of the pre-amplifier and integrator were not designed to operate in this region since the OSCURA experiment requires sub-electron noise operation with N≫40, where the proposed circuit achieves the sensor performance limit.

### 4.2. Parallel Multiplexing while Reading the Next Pixel

The results in previous sections were obtained using serial multiplexing, as shown and explained in Figure 8: the multiplexing occurs at the end of the pixel computation sequence. One of the main contributions of this proposal is to demonstrate the capability of parallel multiplexing. This means piling up the skipper samples of each pixel at the same time that the previous pixel values are being multiplexed and acquired (using S&H as memory) with tested sub-electron noise capabilities. Simultaneous readout and operation of different channels of the sensor is not historically used in CCDs because the large swing (several volts) and fast falling/rising edges of CCD clocks and control signals add noise to the video channels. Control signals and clocks are usually kept steady during integration to obtain charge equivalent noise below micro-volt levels, needed for single-electron event detection [4]. The proposed parallel readout would result in effectively zero added multiplexing time.

**Figure 11:**
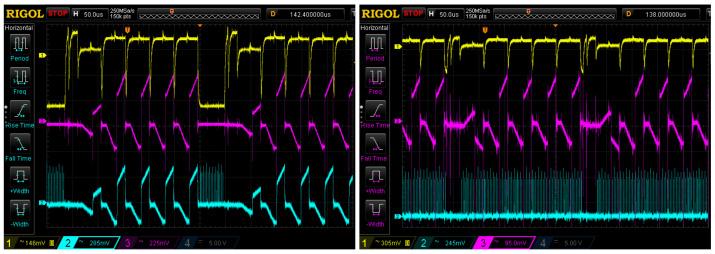
Oscilloscope measurement of waveforms for serial multiplexing (**left**) and parallel multiplexing (**right**) for N=5. Top trace: video signal at the output of the CCD; middle trace: integrator output with 5 sample pile-up; bottom trace: multiplexer output. For the serial readout, the multiplexing takes place after the integration; for the parallel readout, the multiplexing takes place while the next pixel is being read out.

Although, during the time required to measure the *N* pixel samples, it is only necessary to multiplex the previous 16 channels one time, for demonstration purposes, we include additional multiplexing switching during all the periods required to measure the *N* samples to account for a worst-case scenario. In the first pixel sample (out of a total of *N* samples), the final value of the previous pixel is transmitted, and in the other N−1 samples, the multiplexation is repeated, although no samples are acquired by the ADC converter. The oscilloscope measurement in Figure 11 (left) shows the waveforms for serial multiplexing as implemented in previous sections, and parallel multiplexing (right) using N=5. Each oscilloscope capture shows three traces: the top trace shows the video signal at the output of the CCD, the middle trace shows the integrator output with 5 samples piled up and the bottom trace shows the multiplexer output. For the serial readout, the S&H circuit is not used and the output of the multiplexer shows one of the 16 integrators’ output during pixel readout and the multiplexing period afterwards. The parallel readout shows that the multiplexing takes place during all the readout time.

**Figure 12:**
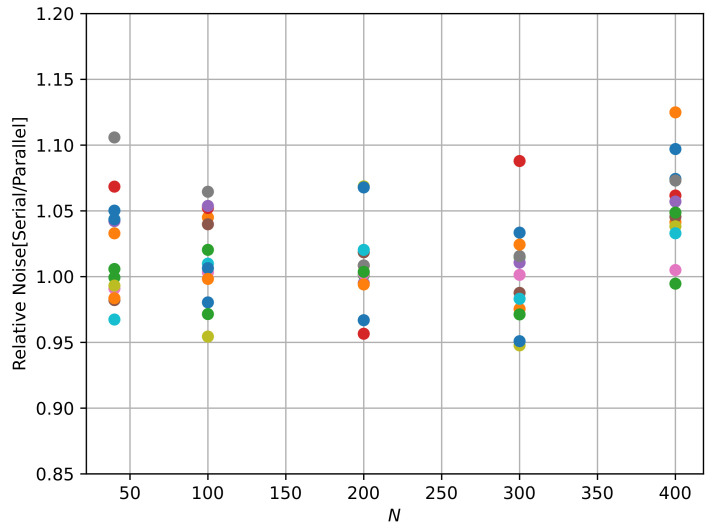
Relative noise: quotient of the noise obtained by serial multiplexing to parallel multiplexing as a function of *N*. For each value of N=40,100,200,300,400, each dot is the relative noise measured in each channel.

To evaluate the relative performance of the serial and parallel multiplexing, Figure 12 shows the quotient of the noise obtained with the serial and parallel multiplexing for a range of *N* values, where the front-end electronics work under $1/\sqrt{N}$ regime. For each value of N=40,100,200,300,400, each dot is the relative noise measured in each channel. The results show that, on average, the value is around 1, indicating that the noise performance of both readout strategies is similar.

**Figure 13:**
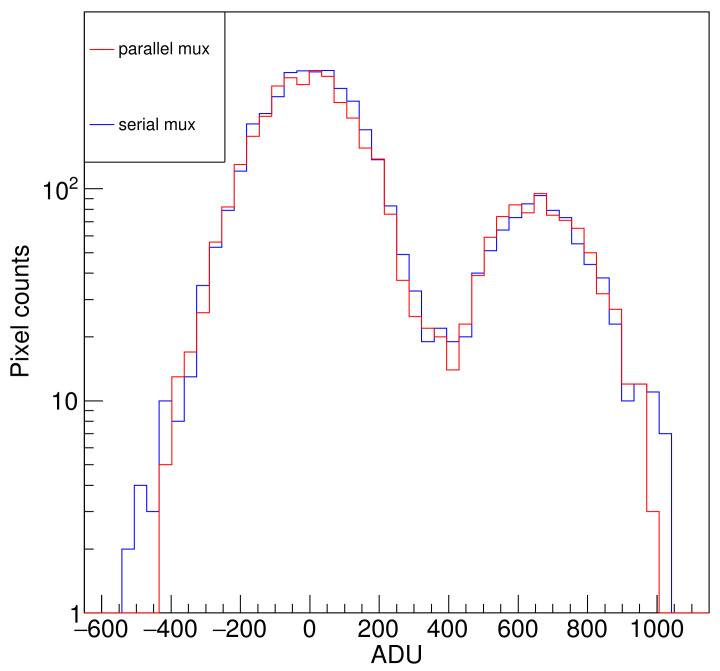
Histogram of zero- and one-electron peaks for serial and parallel multiplexing computed for N=400.

Finally, Figure 13 compares the histogram of the serial and parallel multiplexing for N=400 for one channel. The zero- and one-electron peaks in the histogram show no significant variation in the noise or distributions. This indicates that it is possible to use the S&H to store the pile-up value and multiplex the outputs while reading the next pixel values without affecting the single-electron capability of the sensor.

These results have implications for high-density systems where a lot of sensors need to share ADC inputs. Since, with the parallel scheme, the multiplexing can take place during the actual pixel computation, which extends to a few milliseconds for single-electron-counting CCDs, all the bandwidth requirement can be lowered without adding to the readout time. Referring to the timing diagrams on the oscilloscope capture in Figure 11 (right), 16 channels are multiplexed and acquired approximately during every pixel sample. If N=400 samples per pixel are taken and 16 channels are multiplexed per pixel sample, as a limit, up to 400×16=6400 CCD channels can be read out using a single video input of the LTA without adding any extra delay associated with the multiplexing. Since the LTA has four ADCs, the full OSCURA experiment (24,000 channels) could be instrumented with a single LTA and this novel readout scheme.

## 5. Conclusions

We have presented the architecture and experimental results of a multiplexed readout electronics and strategy suitable for systems with large arrays of particle detectors based on silicon charge-coupled devices (CCDs) with a non-destructive readout output stage. These sensors allow single-electron sensitivity by making several independent measurements of the charge packet. The proposed readout system allows a reduction in the data bandwidth requirement of the systems mentioned by implementing a charge pile-up technique that requires digitization of only the final pixel value instead of the intermediate pixel samples, and it also reduces the number of signals by multiplexing the output of the sensor channels. We experimentally demonstrated that it is possible to maintain the sub-electron noise performance of the sensor while either multiplexing after pixel computation (serial multiplexing) or multiplexing while the next pixel is being computed, implementing a sample and hold circuit as a memory element (parallel multiplexing). Parallel multiplexing enables the architecture to instrument thousands of channels with zero added multiplexing time. For the case shown in the experimental results, up to 6400 Skipper-CCD channels can be read out using a single ADC of the LTA without adding any extra delay associated with the multiplexing. Since the LTA has four ADCs, the full OSCURA experiment (24,000 channels) could be instrumented with a single LTA and this novel readout scheme.

The results encourage the use of the proposed architecture in future experiments with thousands of CCDs and sensitive mass in the order of kilograms for low-threshold particle detection applications.

## Figures and Tables

**Table:** 

**Table:** 

**Table 1:** Comparison between pixel processing methods.

Method	Throughput at ADC Output	Data to Transfer
Fully digital at 15 MSps	270 mbps/channel	858 MB/MPix
Mixed analog/digital DSI	900 kbps/channel	858 MB/MPix
DSI + analog pile-up	2.25 kbps/channel	2.15 MB/MPix

## Data Availability

Not applicable.

## Abbreviations

The following abbreviations are used in this manuscript:

ACQ	Acquisition
ADC	Analog-to-digital converter
ADU	ADC units
CCD	Charge-coupled device
CONNIE	Coherent Neutrino-Nucleus Interaction Experiment
DAQ	Data acquisition
DM	Dark matter
DSI	Double slope integration
LTA	Low-threshold acquisition
OSCURA	Observatory of Skipper-CCDs Unveiling Recoiling Atoms
PCB	Printed circuit board
RMS	Root median square
SENSEI	Sub-Electron-Noise Skipper-CCD Experimental Instrument
S&H	Sample and hold
νIOLETA	Neutrino Interaction Observation with a Low 40 Energy Threshold Array
